# An Erdős-Révész Type Law for the Length of the Longest Match of Two Coin-Tossing Sequences

**DOI:** 10.3390/e27010034

**Published:** 2025-01-03

**Authors:** Karl Grill

**Affiliations:** Institute of Statistics and Mathematical Methods in Economy, TU Wien, Wiedner Hauptstraße 8-10, 1040 Vienna, Austria; karl.grill@tuwien.ac.at

**Keywords:** coin tossing, runs, matching subsequences, strong asymptotics

## Abstract

Consider a coin-tossing sequence, i.e., a sequence of independent variables, taking values 0 and 1 with probability 1/2. The famous Erdős-Rényi law of large numbers implies that the longest run of ones in the first *n* observations has a length $R_{n}$ that behaves like log(n), as *n* tends to infinity (throughout this article, log denotes logarithm with base 2). Erdős and Révész refined this result by providing a description of the Lévy upper and lower classes of the process $R_{n}$. In another direction, Arratia and Waterman extended the Erdős-Rényi result to the longest matching subsequence (with shifts) of two coin-tossing sequences, finding that it behaves asymptotically like 2log(n). The present paper provides some Erdős-Révész type results in this situation, obtaining a complete description of the upper classes and a partial result on the lower ones.

## 1. Introduction

Consider a coin-tossing sequence $\left(X_{n}\right)$, i.e., a sequence of independent random variables satisfying $P\left(X_{n}=0\right)=P\left(X_{n}=1\right)=1/2$. Let $R_{n}$ be the length of the longest head-run, i.e., the largest integer *r* for which there is an *i*, 0≤i≤n−r, for which $X_{i+j}=1$ for j=1,…r. A result of Erdős and Rényi [1] implies that

(1) $$\lim_{n\to \infty }\frac{R_{n}}{\log(n)}=1$$

(throughout this paper, log will denote base 2 logarithms. The notation $\log_{k}$ will be used for its iterates: $\log_{2}\left(x\right)=\log\left(\log\left(x\right)\right)$, $\log_{k+1}\left(x\right)=\log\left(\log_{k}\left(x\right)\right)$. Also, *C* and *c*, with or without an index, are used to denote generic constants that may have different values at each occurrence). The simple result (1) has seen a number of improvements. Erdős and Révész [2] provided a detailed description of the asymptotic behavior of $R_{n}$. In order to formulate their result, let us recall

**Definition 1**  (Lévy classes)**.** *Let $\left(Y_{n}\right)$ be a sequence of random variables. We say that a sequence $\left(a_{n}\right)$ of real numbers belongs to*

- *The upper-upper class of $\left(Y_{n}\right)$ ($UUC(Y_{n})$), if, with probability 1 as n→∞, $Y_{n}\leq a_{n}$ eventually.*
- *The upper-lower class of $\left(Y_{n}\right)$ ($ULC(Y_{n})$), if, with probability 1 as n→∞, $Y_{n}>a_{n}$ for infinitely many n.*
- *The lower-upper class of $\left(Y_{n}\right)$ ($LUC(Y_{n})$), if, with probability 1 as n→∞, $Y_{n}<a_{n}$ for infinitely many n.*
- *The lower-lower class of $\left(Y_{n}\right)$ ($LLC(Y_{n})$), if, with probability 1 as n→∞, $Y_{n}\geq a_{n}$ eventually.*

*Of course, these definitions work best if the sequence $\left(Y_{n}\right)$ obeys some zero-one law.*

Their result is as follows:

Let $\left(a_{n}\right)$ be a nondecreasing integer sequence. Then

- $\left(a_{n}\right)\in UUC\left(R_{n}\right)$ if $\sum_{n}2^{-a_{n}}<\infty$,
- $\left(a_{n}\right)\in ULC\left(R_{n}\right)$ if $\sum_{n}2^{-a_{n}}=\infty$,
- for any ϵ>0, $a_{n}=\left\lfloor \log\left(n\right)-\log_{3}\left(n\right)+\log_{2}\left(e\right)-1+\epsilon \right\rfloor \in LUC\left(R_{n}\right)$,
- for any ϵ>0, $a_{n}=\left\lfloor \log\left(n\right)-\log_{3}\left(n\right)+\log_{2}\left(e\right)-2-\epsilon \right\rfloor \in LLC\left(R_{n}\right)$.

Arratia and Waterman [3] extend Erdős and Rényi’s result in another direction: they consider two independent coin-tossing sequences, $\left(X_{n}\right)$ and $\left(Y_{n}\right)$, and look for the longest matching subsequences when shifting is allowed. Formally, let $M_{n}$ be the the largest integer *m* for which there are i,j with 0≤i,j≤n−m and $X_{i+k}=Y_{j+k}$ for all k=1,…,m. They prove that, with probability 1

(2) $$\lim_{n\to \infty }\frac{M_{n}}{\log(n)}=2.$$

In the present paper, we will make this more precise by providing a description for the upper classes of $\left(M_{n}\right)$ and also some results on its lower classes:

**Theorem 1.**  *Let $\left(a_{n}\right)$ be a nondecreasing integer sequence. We have*

- *$\left(a_{n}\right)\in UUC\left(M_{n}\right)$ if $\sum_{n}n2^{-a_{n}}<\infty$.*
- *$\left(a_{n}\right)\in ULC\left(M_{n}\right)$ if $\sum_{n}n2^{-a_{n}}=\infty$.*
- *for some c, $a_{n}=\left\lfloor 2\log\left(n\right)-\log_{3}\left(n\right)+c\right\rfloor \in LUC\left(M_{n}\right)$.*
- *for some c, $a_{n}=\left\lfloor 2\log\left(n\right)-2\log_{2}\left(n\right)-\log_{3}\left(n\right)+c\right\rfloor \in LLC\left(M_{n}\right)$.*

## 2. Discussion

We leave the proof of Theorem 1 for later and rather discuss some of the concepts that are connected to this problem. One of them is the so-called independence principle: in many, although not all, situations, one may pretend that the waiting times until a given pattern of length *l* is observed have an exponential distribution with parameter $2^{-l}$, and that the waiting times for different patterns are independent. Móri [4] and Móri and Székely [5] provide an account of this principle and its limitations. In our case, all results but the lower-lower class one are more or less in tune with this principle.

Another question that is closely related is that of the number N(n,l) of different length *l* subsequences of $\left(X_{1},\ldots ,X_{n}\right)$. This question does not seem to have been considered by literature very much; there is one remarkable result by Móri [6]: in the remark following the statement of Theorem 3 in that paper, he mentions that with probability one for large *n*, the largest *l* for which all $2^{l}$ possible patterns occur as subsequences of $\left(X_{1},\ldots ,X_{n}\right)$ is either $\left\lfloor \log\left(n\right)-\log_{2}\left(n\right)-\epsilon \right\rfloor$ or $\left\lfloor \log\left(n\right)-\log_{2}\left(n\right)+\epsilon \right\rfloor$ for any ϵ>0. The independence principle would suggest that N(n,log(n))/n is bounded away from 0 with probability one, and this or even the less stringent $N\left(n,\log\left(n\right)\right)\geq n{\left(\log_{2}\left(n\right)\right)}^{-c}$ for large *n* would be an important step towards removing the double log term from the LLC result, as we conjecture that, for some c>0, $\log\left(n\right)-c\log_{3}\left(n\right)\in LLC\left(M_{n}\right)$. Unfortunately, we are only able to obtain N(n,log(n))≥cn/log(n), which is also implied by Móri’s result.

## 3. Proofs

**Proof of the upper-upper class result.**  Both upper class statements are fairly easy to prove. First, observe that under our assumptions, the convergence of

(3) $$\sum_{n=1}^{\infty }n2^{-a_{n}}$$

is equivalent to that of

(4) $$\sum_{k=1}^{\infty }n_{k}^{2}2^{-a_{n_{k}}}$$

with $n_{k}=2^{k}$.

Now, define events

(5) $$A_{k}=\left[M_{n_{k}}\geq a_{n_{k-1}}\right].$$

$A_{k}$ occurs if in one of the ${\left(n_{k}+1-a_{n_{k-1}}\right)}^{2}$ pairs of sequences

(6) $$(\left(X_{i+1},\ldots ,X_{i+a_{k-1}}\right),\left(\left(Y_{j+1},\ldots ,Y_{j+a_{k-1}}\right)\right)$$

both sequences agree. That provides the trivial upper bound

(7) $$P\left(A_{k}\right)\leq n_{k}^{2}2^{-a_{n_{k-1}}},$$

so, by our assumptions, $\sum_{n}P\left(A_{k}\right)<\infty$, and the Borel-Cantelli Lemma implies that, with probability 1, only finitely many events $A_{k}$ occur. Thus, for sufficiently large *k*, $M_{n_{k}}\leq a_{n_{k-1}}$, and for $n_{k-1}\leq n\leq n_{k}$, we have

(8) $$M_{n}\leq M_{n_{k}}\leq a_{n_{k-1}}\leq a_{n}.$$

This shows that $\left(a_{n}\right)\in UUC\left(M_{n}\right)$, as claimed. □

**Proof of the upper-lower class result.**  We may assume without loss of generality that $n^{2}2^{-a_{n}}\leq 1/4$.

Again, let $n_{k}=2^{k}$. We want to use the second Borel-Cantelli Lemma, so we are defining independent events

(9) $$A_{k}=\left[\exists i,j:n_{k-1}<i,j\leq n_{k}:X_{i+s}=Y_{j+s},s=0,\ldots ,a_{n_{k}}-1\right]$$

This is the union of the events

(10) $$B_{ij}=\left[X_{i+s}=Y_{j+s},s=0,\ldots ,a_{n_{k}}-1\right]$$

with $n_{k-1}<i,j\leq n_{k}$. We endow the set of pairs (i,j) with the lexicographic order. For a subset *I* of the integers, Bonferroni’s inequality provides

(11) $$P\left(\underset{(i,j)\in I\times I}{⋃}B_{ij}\right)\geq \sum_{(i,j)\in I\times I}P\left(B_{ij}\right)-\sum_{\left(i,j\right),\left(i^{\prime },j^{\prime }\right)\in I\times I,\left(i,j\right)<\left(i^{\prime },j^{\prime }\right)}P\left(B_{ij}\cap B_{i^{\prime }j^{\prime }}\right).$$

Let $d\left(\left(i,j\right),\left(i^{\prime },j^{\prime }\right)\right)=\max(|i-i^{\prime }|,|j-j^{\prime }\left|\right)$. If $d\left(\left(i,j\right),\left(i^{\prime },j^{\prime }\right)\right)\geq a_{n_{k}}$, then $P\left(B_{ij}\cap B_{i^{\prime }j^{\prime }}\right)=2^{-2a_{n_{k}}}$, otherwise $P\left(B_{ij}\cap B_{i^{\prime }j^{\prime }}\right)=2^{-(a_{n_{k}}+d\left(\left(i,j\right),\left(i^{\prime },j^{\prime }\right)\right)}$.

Let $I=\{i:n_{k-1}<i\leq n_{k}:4|i\}$. Using this in (11) yields the value ${\left|I\right|}^{2}2^{-a_{n_{k}}}$ for the first sum. In the second sum, for given (i,j) and $d<a_{n_{k}}/4$, there are no more than 2(2d+1) pairs $\left(i^{\prime },j^{\prime }\right)$ with $d(\left(i,j\right),\left(i^{\prime },j^{\prime }\right))=4d$. The number of pairs of pairs $(\left(i,j\right),\left(i^{\prime },j^{\prime }\right)$ with $d\left(\left(i,j\right),\left(i^{\prime },j^{\prime }\right)\right)\geq a_{n_{k}}$ is trivially bounded by ${\left|I\right|}^{4}/2$. Putting these together, we arrive at the upper estimate

(12) $${\left|I\right|}^{2}2^{-a_{n_{k}}}(\sum_{d=1}^{\infty }\left(2d+1\right)2^{1-4d}+{\left|I\right|}^{2}2^{-1-a_{n_{k}}})<\frac{1}{2}{\left|I\right|}^{2}2^{-a_{n_{k}}}.$$

In total,

(13) $$P\left(A_{k}\right)\geq \frac{1}{2}{\left|I\right|}^{2}e^{-a_{n_{k}}}=\frac{1}{128}n_{k}^{2}2^{-a_{n_{k}}},$$

keeping in mind that $\left|I\right|=n_{k}/8$.

and $\sum_{k}P\left(A_{k}\right)=\infty$. Borel-Cantelli implies that, with probability 1, infinitely many events $A_{k}$ occur. Thus, for infinitely many *k*, $M_{n_{k}}\geq a_{n_{k}}$, so $\left(a_{n}\right)\in ULC\left(M_{n}\right)$. □

For the lower class results, we first prove some lemmas:

**Lemma 1.**  *With probability *1* for n sufficiently large*

(14) $$\frac{n}{4\log(n)}\leq N\left(n,\left\lfloor \log\left(n\right)\right\rfloor \right)\leq n$$

**Proof of Lemma 1.**  The lower part is a direct consequence of Móri’s result: as for sufficiently large *n* $N\left(n,\left\lfloor \log\left(n\right)-\log_{2}\left(n\right)-1\right\rfloor \right)=2^{\left\lfloor \log\left(n\right)-\log_{2}\left(n\right)-1\right\rfloor }\geq \frac{n}{4\log(n)}$ and, obviously $N\left(n,\left\lfloor \log\left(n\right)\right\rfloor \right)\geq N\left(n,\left\lfloor \log\left(n\right)-\log_{2}\left(n\right)-1\right\rfloor \right)-\log_{2}\left(n\right)-2$, as extending two different sequences from length $\left\lfloor \log\left(n\right)-\log_{2}\left(n\right)\right\rfloor$ to ⌊log(n)⌋ keeps them different; it can only happen that some of them are extended beyond index *n*, but this can affect at most $\log_{2}\left(n\right)+2$ of them. □

**Lemma 2.**  *Let S be a set of $m<2^{l}$ sequences of length l<n, and let A be the event that none of the sequences in S occurs as a subsequence of $\left(X_{1},\ldots ,X_{n}\right)$. For $l\leq n^{\prime }<n$, let B be the event that some sequence from S is a subsequence of $X_{1},\ldots ,X_{n^{\prime }}$. Then*

(15) $$\left({\left(1-P\left(B\right)\right)}^{2}-\left\lfloor n/n^{\prime }\right\rfloor m2^{-l}\right){\left(1-P\left(B\right)\right)}^{\left\lfloor n/n^{\prime }\right\rfloor }\leq P\left(A\right)\leq {\left(1-P\left(B\right)\right)}^{\left\lfloor n/n^{\prime }\right\rfloor }.$$

**Proof of Lemma 2, upper part.**  Consider the $\left\lfloor n/n^{\prime }\right\rfloor$ sequences $X_{kn^{\prime }+1},\ldots ,X_{\left(k+1\right)n^{\prime }}$ for $k=0,\ldots \lfloor n/n^{\prime }\rfloor -1$. Each of these has probability 1−P(B) that it does not contain a subsequence that lies in *S*, and by independence

(16) $$P\left(A\right)\leq {\left(1-P\left(B\right)\right)}^{\left\lfloor n/n^{\prime }\right\rfloor }.$$

□

**Proof of Lemma 2, lower part.**  Assume for simplicity that *n* is a multiple of $n^{\prime }$, say $n=Nn^{\prime }$. Again, we split $\left(X_{1},\ldots ,X_{n}\right)$ into *N* blocks of length $n^{\prime }$, and the probability that none of those contains a sequence from *S* is

(17) $${\left(1-P\left(B\right)\right)}^{N}.$$

It can still happen that there is a sequence from *S* that crosses one of the boundaries between the blocks. There are N−1 boundaries, and for each of those, there are l−1 possible subsequences of length *l* crossing it. The probability that this one is from *S* but none of the *N* blocks contains one can be estimated above by the probability that it is from *S* and none of the N−2 blocks not adjacent to it contains one from *S*. This provides the upper bound

(18) $$\left(N-1\right)\left(l-1\right)m2^{-l}{\left(1-P\left(B\right)\right)}^{N-2}$$

for the probability that there is a subsequence from *S* in $\left(X_{1},\ldots ,X_{n}\right)$ but none in any of the *N* blocks. Subtracting this from (17), we obtain the lower bound

(19) $$\left({\left(1-P\left(B\right)\right)}^{2}-\left(N-1\right)\left(l-1\right)m2^{-l}\right){\left(1-P\left(B\right)\right)}^{N-2},$$

and the general case is obtained by observing that the probability P(A) for a given *n* is bounded below by the one that we get for $n^{\prime }\left\lceil n/n^{\prime }\right\rceil$. □

In this lemma, the lower and upper bounds are rather close. Its applicability, however, depends on the availability of good estimates for the probability P(B). There is the trivial upper bound $n^{\prime }m2^{-l}$ and an almost as simple lower bound $\mathrm{const}.n^{\prime }ml^{-1}2^{-l}$ (given $n^{\prime }ml^{-1}2^{-l}<1$). Bridging the gap between these requires deeper insight into the structure of *S*.

**Proof of the lower-lower class result.**  In both the lower-lower and lower-upper parts, we consider the asymptotics of the longest match found between $\left(X_{1},\ldots ,X_{n}\right)$ and $\left(Y_{1},\ldots ,Y_{n}\right)$ under the condition that the sequence $\left(Y_{n},n\in N\right)$ is given. Doing so, we may assume that $Y=(Y_{n},n\in N)$ is a “typical” coin-tossing sequence, in the sense that it possesses some property that holds with probability 1. In the sequel, all probabilities are understood as conditional with respect to such a typical sequence Y. For the lower-lower class result, we let $n=n_{l}=\left\lceil C2^{l/2}l\sqrt{\log(l)}\right\rceil$, $n^{\prime }=n_{l}^{\prime }=l$, and $m=m_{l}=\left\lceil \frac{n}{4\log(n)}\right\rceil$ in Lemma 2. Clearly, as $\left(X_{1},\ldots ,X_{l}\right)$ only has one length *l* subsequence, P(B) equals $\tilde{m}2^{-l}$, where $\tilde{m}$ is the number of different sequences of length *l* in $Y_{1},\ldots ,Y_{n_{l}}$. For sufficiently large *l*, $\tilde{m}\geq m_{l}$ by Lemma 1, and we obtain an upper estimate

(20) $$p_{l}=\exp\left(-m\left\lfloor n/l\right\rfloor 2^{-l}\right)=\exp\left(-\frac{1}{2}C^{2}\log\left(l\right)\left(1+o\left(1\right)\right)\right)$$

for the probability (conditional on Y) that there is no match of length *l* between $\left(Y_{1},\ldots ,Y_{n_{l}}\right)$ and $\left(X_{1},\ldots ,X_{n_{l}}\right)$. For $C>\sqrt{2/\log(e)}$, the series $\sum_{l}p_{l}$ converges, so with probability 1, we have $M(n_{l})\geq l$ for all but finitely many *l*. Thus, for $n_{l-1}\leq n<n_{l}$, $M_{n}\geq M_{n_{l-1}}\geq l-1$. Inverting the relationship between $n_{l}$ and *l* yields $l=2\log\left(n_{l}\right)-2\log_{2}\left(n_{l}\right)-\log_{3}\left(n_{l}\right)+O\left(1\right)$, so, for some constant *c* and *l* large enough, we obtain $M_{n}\geq l-1\geq 2\log\left(n\right)-2\log_{2}\left(n\right)-\log_{3}\left(n\right)-c$, which proves the lower-lower class result. □

**Proof of the lower-upper class result.**  This time, we need to make our choice of the parameters in Lemma 2 with a little more sophistication. We start with $l=l_{k}=k^{2}$ for k∈N. Then, $n=n_{k}=\left\lfloor C2^{l_{k}/2}\sqrt{\log(l_{k})}\right\rfloor \prime$. As the set *S*, we choose the set $S_{k}$ of all sequences of length $l_{k}$ contained in $\left(Y_{1},\ldots ,Y_{n_{k}}\right)$. $n^{\prime }=n_{k}^{\prime }$ is chosen in such a way that $m_{k}n_{k}^{\prime }2^{-l_{k}}\to 0$ and $m_{k}n_{k}l_{k}2^{-l_{k}}/n_{k}^{\prime }\to 0$, $n_{k}^{\prime }=\left\lfloor 2^{l_{k}/4}\right\rfloor$ is a possible choice.

We define the events

(21) $$A_{k}=\left\{\mathrm{There is no sequence from}\;S_{k}\;\mathrm{in}\left(X_{1},\ldots X_{n_{k}}\right)\right\},$$

(22) $${\tilde{A}}_{k}=\left\{\mathrm{There is no sequence from}\;S_{k}\;\mathrm{in}\left(X_{n_{k-1}+1},\ldots X_{n_{k}}\right)\right\}$$

(23) $$B_{k}=\left\{\mathrm{There is a sequence from}\;S_{k}\;\mathrm{in}\left(X_{1},\ldots X_{n_{k}^{\prime }}\right)\right\}$$

(this last is just the event *B* from Lemma 2).

Lemma 2 gives us

(24) $$P\left(A_{k}\right)={\left(1-P\left(B_{k}\right)\right)}^{\left\lfloor n_{k}/n_{k}^{\prime }\right\rfloor }\left(1+o\left(1\right)\right)$$

and

(25) $$P\left({\tilde{A}}_{k}\right)={\left(1-P\left(B_{k}\right)\right)}^{\left\lfloor \left(n_{k}-n_{k-1}\right)/n_{k}^{\prime }\right\rfloor }\left(1+o\left(1\right)\right).$$

The trivial estimate $P\left(B_{k}\right)\leq m_{k}n_{k}^{\prime }2^{-l_{k}}\leq n_{k}^{2}2^{-l_{k}}$ yields

(26) $$P\left(A_{k}\right)\geq e^{-2C^{2}\log\left(k\right)\left(1+o\left(1\right)\right)},$$

which diverges if we choose $C<1/\sqrt{2\log(e)}$.

We are going to use the Borel-Cantelli Lemma in the usual form for dependent events:

**Lemma 3**  (Borel-Cantelli II)**.** *If the sequence $\left(A_{n}\right)$ satisfies*

(27) $$\sum_{n\in N}P\left(A_{n}\right)=\infty$$

*and*

(28) $$\lim_{n\to \infty }\frac{\sum_{i=1}^{n}\sum_{j=1}^{n}P\left(A_{i}\cap A_{j}\right)}{{\left(\sum_{i=1}^{n}P\left(A_{i}\right)\right)}^{2}}=1,$$

*then*

(29) $$P(\underset{n}{lim sup}A_{n})=1,$$

To this end, we need an upper bound for $P(A_{i}\cap A_{j})$ for i<j. We have

(30) $$P\left(A_{i}\cap A_{j}\right)\leq P\left(A_{i}\cap {\tilde{A}}_{j}\right)=P\left(A_{i}\right)P\left({\tilde{A}}_{j}\right).$$

By our Equations (24) and (25) from above

$$P\left({\tilde{A}}_{j}\right)=P\left(A_{j}\right){\left(1-P\left(B_{j}\right)\right)}^{-n_{j-1}/n_{j}^{\prime }}\left(1+o\left(1\right)\right)=P\left(A_{j}\right)e^{m_{j}n_{j-1}2^{-l_{j}}\left(1+o\left(1\right)\right)}=$$

(31) $$P\left(A_{j}\right)\left(1+o\left(1\right)\right),$$

as $n_{j-1}\leq n_{j}e^{1-2j}$ and $m_{j}n_{j}2^{-l_{j}}\leq n_{j}^{2}2^{-l_{j}}=O\left(\log j\right)$.

This means that for any ϵ>0, there is a number $j_{0}$ such that, for $j>j_{0}$ and i<j, the inequality

(32) $$P\left(A_{i}\cap A_{j}\right)\leq \left(1+\epsilon \right)P\left(A_{i}\right)P\left(A_{j}\right)$$

holds. Plugging this into

(33) $$\sum_{j=1}^{n}\sum_{i=1}^{n}P\left(A_{i}\cap A_{j}\right)=\sum_{i=1}^{n}P\left(A_{i}\right)+2\sum_{j=2}^{n}\sum_{i=1}^{j-1}P\left(A_{i}\cap A_{j}\right)$$

yields the estimate

(34) $$\sum_{j=1}^{n}\sum_{i=1}^{n}P\left(A_{i}\cap A_{j}\right)\leq \sum_{i=1}^{n}P\left(A_{i}\right)+j_{0}^{2}+\left(1+\epsilon \right){\left(\sum_{i=1}^{n}P\left(A_{i}\right)\right)}^{2}.$$

As $\sum_{i=1}^{\infty }P\left(A_{i}\right)=\infty$, we get

(35) $$\underset{n\to \infty }{lim sup}\frac{\sum_{i=1}^{n}\sum_{j=1}^{n}P\left(A_{i}\cap A_{j}\right)}{{\left(\sum_{i=1}^{n}P\left(A_{i}\right)\right)}^{2}}\leq 1+\epsilon .$$

As ϵ>0 is arbitrary, the sequence $\left(A_{k}\right)$ satisfies the assumptions of Lemma 3, so, with probability 1, infinitely many of the events $\left(A_{k}\right)$ occur. Thus, with probability 1 for infinitely many *k*, $M_{n_{k}}<l_{k}$. Observing that $l_{k}=2\log\left(n_{k}\right)-\log_{3}\left(n_{k}\right)+O\left(1\right)$, we obtain our lower-upper class result. □

## Data Availability

No new data were created or analyzed in this study. Data sharing is not applicable.

## References

[1] Erdős P., Rényi A. (1970). On a new law of large numbers. J. Anal. Math..

[2] Erdős P., Révész P., Ciszár I., Elias P. (1977). On the length of the longest head-run. Topics in Information Theory.

[3] Arratia R., Waterman S. (1985). An Erdős-Rényi law with shifts. Adv. Math..

[4] Móri T. (1985). Large deviation results for waiting times in repeated experiments. Acta Math. Hung..

[5] Móri T., Székely G. (1984). Asymptotic independence of pure head stopping times. Stat. Probab. Lett..

[6] Móri T. (1991). On the waiting time till each of some given patterns occurs as a run. Probab. Theory Relat. Fields.

